# Editorial: Microbiota homeostasis and metabolic reprogramming in cancer development and digestive diseases

**DOI:** 10.3389/fcell.2025.1653798

**Published:** 2025-07-22

**Authors:** Kai Li

**Affiliations:** Division of Abdominal Tumor Multimodality Treatment, Cancer Center and Lab of Experimental Oncology, State Key Laboratory of Biotherapy, West China Hospital, Sichuan University, Chengdu, Sichuan, China

**Keywords:** microbiota, metabolic reprogramming, cancer development, tumor microenvironment, therapeutic resistance

## Glucose metabolism and multidrug resistance

Wang et al. review how glucose metabolism fuels multidrug resistance (MDR) in cancer. They describe that the aberrant relocation of key glycolytic enzymes such as PFKP, PKM2 and PGK1 to the nucleus, membrane, or mitochondria, which endows glycolytic enzymes with novel and non-classical pro-oncogenic functions, accelerates increased drug efflux via ATP-binding cassette transporters and undermines chemotherapeutic efficacy. Compensatory upregulation of other macromolecular metabolism, including purine pyrimidine, fatty acid, and glutamine, protects tumor cells against small molecule inhibitors targeting glucose metabolism, highlighting the dynamic crosstalk and complexity of tumor metabolic plasticity that enables tumor cells to survive under drug pressure and adapts MDR phenotypes. These findings suggest that combination therapy with multiple drugs for different targets will effectively eliminate tumor cells and simultaneously decrease tolerance, off-targets, and adverse effects.

## Tumor-associated neutrophil metabolism and immunotherapy

Lin et al. systematically summarize how tumor-associated neutrophils (TANs) undergo metabolic reprogramming within the hypoxic, nutrient-deprived tumor microenvironment (TME), focusing on the alterations in glycolysis, lipid metabolism, and amino acid pathways. They emphasize that TANs upregulate glycolytic enzymes and increase lactate production to fuel pro-tumor functions and metastatic support; meanwhile, enhanced fatty acid oxidation and lipid uptake provide necessary energy under stress conditions, and glutamine and arginine metabolism are rewired to drive TAN polarization toward immunosuppressive (N2) or cytotoxic (N1) phenotypes. This functional plasticity and heterogeneity, which are dynamically regulated by a variety of cytokines and chemokines secreted from tumor cells and stromal cells within the TME, facilitate immune evasion and therapeutic resistance. Targeting the unique metabolic nodes offers a promising route to restore TAN’s antitumor activity and enhance immunotherapy efficacy.

## Microbiota and thyroid hormone metabolism

Cadena-Ullauri et al. explore how gut dysbiosis in thyroid cancer patients alters thyroid hormone homeostasis by disrupting iodine uptake and thyroxine-to-triiodothyronine conversion. Reduction of *Lactobacillus* and Bifidobacterium diminishes short-chain fatty acids (SCFA) generation and impairs intestinal barrier integrity, leading to reduced sodium-iodide symporter (NIS) expression and iodine absorption. Gut microbiota-derived endotoxins and exotoxins enables to alter thyroid hormone metabolism by directly regulating the activity of thyroid hormone synthesis-related enzymes. Importantly, gut microbiota dysbiosis triggers intestinal inflammation and oxidative stress, which facilitates the pathogenesis of autoimmune thyroid diseases and thyroid cancer. This review highlights validated alterations in microbial taxa and enzymatic activities during thyroid cancer progression, suggesting that probiotic supplementation or microbiota-targeted interventions could restore NIS function and normalize triiodothyronine/thyroxine levels in thyroid cancer patients.

## Gut microbiota in colorectal liver metastasis

Liu et al. propose a framework in which harmful gut microbes in colorectal cancer (CRC) patients impede the intestinal barrier and release high levels of their own metabolites—such as bile acids, SCFA, lipopolysaccharide, and indole derivatives—into the circulation. These compounds drive chronic inflammation within the tumor microenvironment and ultimately enhance immunosuppression, while also directly stimulating cancer cell proliferation. Moreover, barrier disruption permits these pathogenic microbes to translocate and colonize distant metastatic sites via the gut-liver axis, where they exert additional pro-tumor functions. The liver, as an immunologically “cold” organ, provides a unique microenvironment that compromises the overall immune response and the effectiveness of immune checkpoint inhibitors. Thus, liver metastasis has been considered a critical reason in immunotherapy failure in metastatic CRC. This model highlights specific microbial metabolites and barrier integrity as critical intervention points for disrupting the cancer-promoting feedback loop.

## Microbiota–metabolism crosstalk in cancer progression

Li et al. provide a comprehensive overview of how the microbiota—through its secondary metabolites—drives metabolic reprogramming across diverse tumor types, including gastric, colorectal, lung, breast, and head and neck squamous cell carcinomas, thereby promoting disease progression. They systematically analyze how these microbially mediated metabolic alterations fuel tumor growth and advance malignancy, propose a therapeutic paradigm centered on targeting tumor-associated microbiota and their metabolic outputs, and concisely evaluate the efficacy of relevant pharmacological interventions. Personalized treatments targeting identified metabolic–microbial axes may offer novel and beneficial therapeutic strategies.

## Anesthetics and gynecological cancer

Cheng et al. review how perioperative anesthetic agents influence cancer cell metabolism and immune function via sympathetic nervous system and hypothalamic-pituitary-adrenal axis in gynecological cancers. Although local anesthetics (ropivacaine and lidocaine) and intravenous anesthetics (dexmedetomidine and propofol) have been demonstrated to exhibit anti-tumor activity in gynecological malignancies, most volatile anesthetics possess paradoxically dual effects on tumorigenesis and development. Optimizing anesthesia protocols (such as regional blocks or specific drug combinations) will provide safer and more efficient regimens to minimize pro-tumor influences, decline recurrence and improve the prognosis of patients with gynecological cancers.

Overall, this Research Topic provides comprehensive and in-depth insights of the complex relationship between tumor-associated microbiota homeostasis and the corresponding metabolic reprogramming in various cancers, while highlighting the therapeutic potential of targeting the microbiota—particularly in tumor types that are highly sensitive to metabolic interventions. Notably, most current studies emphasize how dysbiosis drives tumor progression, but there is a paucity of research on whether tumor-induced changes in the microenvironment further exacerbate this imbalance—an important avenue for future investigation.

